# Coevolution of *Drosophila melanogaster* mtDNA and Wolbachia Genotypes

**DOI:** 10.1371/journal.pone.0054373

**Published:** 2013-01-17

**Authors:** Yury Ilinsky

**Affiliations:** 1 Laboratory of Populations Genetics, Institute of Cytology and Genetics of Siberian Branch of Russian Academy of Sciences, Novosibirsk, Russia; 2 Department of Cytology and Genetics, Novosibirsk State University, Novosibirsk, Russia; Centro de Pesquisas René Rachou, Brazil

## Abstract

Maternally inherited microorganisms can influence the mtDNA pattern of variation in hosts. This influence is driven by selection among symbionts and can cause the frequency of mitochondrial variants in the population to eventually increase or decrease. *Wolbachia* infection is common and widespread in *Drosophila melanogaster* populations. We compared genetic variability of *D. melanogaster* mitotypes with *Wolbachia* genotypes among isofemale lines associated with different geographic locations and time intervals to study coevolution of the mtDNA and *Wolbachia*. Phylogenetic analysis of *D. melanogaster* mtDNA revealed two clades diverged in Africa, each associated with one of the two *Wolbachia* genotype groups. No evidence of horizontal transmission of *Wolbachia* between maternal lineages has been found. All the mtDNA variants that occur in infected isofemale lines are found in uninfected isofemale lines and vice versa, which is indicative of a recent loss of infection from some maternal fly lineages and confirms a significant role of *Wolbachia* in the *D. melanogaster* mtDNA pattern of variation. Finally, we present a comparative analysis of biogeographic distribution of *D. melanogaster* mitotypes all over the world.

## Introduction

*Wolbachia* is a genus of maternally transmitted endosymbiotic bacteria that is found in a wide range of arthropods and nematodes [1]–[3]. The effects of *Wolbachia* on their hosts are quite diverse, including mutualism and reproductive parasitisms such as cytoplasmic incompatibility, parthenogenesis, male-killing, feminization, which can provide a reproductive advantage to infected females [4].

*Wolbachia* infection is common and widespread in *Drosophila melanogaster* [5]–[12]. The frequency of infected individuals in populations normally ranges from 10% to 90% and is on average about 50% per population. However, some populations have an extremely low frequency of infected individuals, e.g. a population from West Africa sampled in 2010, that had only one infected individual in a sample of over hundred flies [11].

In *D. melanogaster*, *Wolbachia* occurs as a single strain named wMel. This conclusion was made after different gene sequences were found monomorphic [13]–[16]; however, this strain was further subdivided into several genotypes (wMelCS, wMelCS2, wMel, wMel2, wMel3) by using genetic markers such as inversion, variable number tandem repeats (VNTRs) and transposon insertion sites [7]. The wMel and wMelCS genotypes are found all over the world; however, the wMel genotype is most prevalent [7]–[10]. The other *Wolbachia* genotypes are rare and local.

Selection on maternally inherited symbionts can lead to changes in the mtDNA haplotype frequency in host populations (indirect selection on mtDNA variation). Hurst and Jiggins [17] classify the influence of microoganisms on mitochondrial diversity into four types: 1) symbiont-driven reduction in mtDNA diversity, 2) symbiont-driven increase in mtDNA diversity, 3) symbiont-driven change in mtDNA variation over space and 4) symbiont-associated mtDNA paraphyly. Facts about *Wolbachia*’s influence on mtDNA diversity are numerous, with many of them reviewed by Hurst and Jiggins [17], where they also suggested further studies in terms of evolutionary history of *Wolbachia* hosts [10], [12], [18]–[21].

Several attempts have been made to find a link between mitochondrial DNA diversity in *D. melanogaster* and *Wolbachia*. Solignac et al. [5] compared the infection status and restriction-site polymorphism of *D. melanogaster* mtDNA and came to the conclusion that “cytoplasm infection is irrespective of mtDNA haplotypes”. Nunes et al. [7] performed a more detailed comparison of partial *cox1* sequences for *D. melanogaster* with *Wolbachia* genotypes and concluded that “the *Wolbachia* infection was not randomly distributed among flies with different mtDNA haplotypes”. Previously, we used a similar approach and came to the conclusion that there were three haplotypes of the *D. melanogaster* mitochondrial *cox1* gene, each associated with one of the three *Wolbachia* genotypes [8], [22]. Here we present a more extensive phylogenetic analysis of 2757-bp and 1280-bp of mtDNA from *D. melanogaster* isofemale lines harboring as many *Wolbachia* genotypes as have been found in wild populations. We demonstrate a perfect consistency between major mitochondrial lineages and *Wolbachia* genotypes, which suggests an absence of *Wolbachia* horizontal transmission among *D. melanogaster* lineages or if such events exist then there is no conspicuous effect on the cytotype patterns. Similar results have been obtained recently by Richardson et al. [12], with a different sample of strains used. We extend our results and those of [12] to provide a comparative analysis of both datasets on the diversity and biogeography mitotypes and *Wolbachia* genotypes in *D. melanogaster*.

## Methods and Materials

### Fly Lines

A total of 413 samples for mitochondrion polymorphism are used. There are such datasets: a) 62 stocks were sequenced by me in range 502–2757 bp mtDNA fragment (Table 1), b) 8 mitochondrial genomes present in GenBank sequenced by other authors (Table 1), c) 25 stocks tested for 37C/T polymorphism (see below), d) 28 sequences of 1515 bp fragment taken from Rand et al. [23]; e) 290 sequences of 2757 bp fragment derived from Richardson et al. [12]. Most of lines in dataset “a” are from the Laboratory of Populations Genetics of the Institute of Cytology and Genetics of the Siberian Branch of the Russian Academy of Sciences. Lines **10030** and **10032** infected with wMel2 were courtesy of Masayoshi Watada (Ehime University, National Bio-Resource Project in Japan), **w^1118^** infected with wMelPop [24] was courtesy of Elena Kiseleva (Institute of Cytology and Genetics, Russia). Dataset “b” also includes the 12508 bp sequence of **w^1118^** stock produced by Clancy [25] that is indicated in footnotes of Table 1 because of the identity of Clancy’s and my results in the compatible region. The “d” dataset represents lines derived from populations of Africa, Europe, Asia, North and South America. The “e” dataset contains information on samples from a single population of Northern America [26], Europe, populations of Africa [27] and a chimerical sequence – NC001709 that is composed from **Canton-S** and **Oregon-R** stocks [12], [28]–[30].

**Table 1:** The *Drosophila melanogaster* isofemale lines used.

Infection status	Name or number of the lines	Origin, location and year of sampling oforiginal flies	GenBank accession number	size (bp)	10C/T-37C/T mitotype
wMel genotype	3110	Zvenigorodka, Ukraine, 2003	JF736855	2757	CT
	U4	Uman, Ukraine, 2004	JF736845	2757	CT
	Bi90	Bishkek, Kyrgyzstan, 2004	JF736853	2757	CT
	s400	Sochi, Caucasus, Russia, 2004	JF736848	2757	CT
	Harwich	Harwich, Massachusetts, USA, 1967	JF736865	2757	CT
	335	Chemal, Altai, Russia, 2003	JF736854	2757	CT
	2–37	LS, Russia, early 1970	JF736858	2757	CT
	11-Sinai	Sinai Peninsula, Egypt, 2010	JF781531	2757	CT
	90172	Uman, Ukraine, 1990	JN052155	1280	CT
	90084	Uman, Ukraine, 1990	JN052152	1280	CT
	U10	Uman, Ukraine, 2004	JN052157	1280	CT
	U84-1-26	Uman, Ukraine, 1984	JF730694	502	CT
	10 isofemale lines	Uman, Ukraine, 1990	JF730694	502	CT
	611Sin	Sinai Peninsula, Egypt, 2010	JF730694	502	CT
wMel2 genotype	10030	Amamioshima, Japan, 1982	JF736856	2757	CT
	10032	Amamioshima, Japan, 1982	JF736857	2757	CT
wMel4 genotype	12-Sin	Sinai Peninsula, Egypt, 2010	JF736866	2757	CT
wMelCS genotype	Canton-S	Canton, Ohio, USA, 1930	JQ416156	2757	TC
	921189	Biysk, Altai, Russia, 1992	JF736847	2757	TC
	w^1118^	wMelPop-infected LS	JF736852*	2757	TC
	w153	Tashkent, Uzbekistan, 1989	JF736849	2757	TC
	3–1	Uman, Ukraine, 1971	JF736867	2757	TC
wMelCS2 genotype	109	Kishinev, Moldavia, 1984	JF736850	2757	CC
	181	Tbilisi, Georgia, 1989	JF736851	2757	CC
	w75	Gomel, Belorussia, 1980	JF736846	2757	CC
	2–23	LS, Russia, early in 1976	JF736864	2757	CC
	88233	Uman, Ukraine, 1988	JN052151	2604	CC
	90776	Dushanbe, Tajikistan, 1990	JN052159	1280	CC
	93220	Biysk, Altai, Russia, 1993	JN052158	1280	CC
uninfected	Oregon-R**	Roseburg, Oregon, USA, 1925	AF200828	14905	TC
	921151	Biysk, Altai, Russia, 1992	JF736863	2757	CC
	w36	Krasnodar, Russia, 1978	JF736859	2757	TC
	w77	Tashkent, Uzbekistan, 1981	JF736861	2757	CC
	w166	Ulan-Ude, Buryatia, Russia, 1988	JF736862	2757	TC
	w59	Berlin, German, 1988	JF736860	2757	TC
	U84-3	Uman, Ukraine, 1984	JN052150	1280	TC
	90021	Uman, Ukraine, 1990	JN052153	1280	TC
	90163	Uman, Ukraine, 1990	JN052156	1280	CT
	90187	Uman, Ukraine, 1990	JN052154	1280	CT
	6 isofemale lines	Uman, Ukraine, 1984	JF730694	502	CT
	88332	Uman, Ukraine, 1988	JF730696	502	CC
	90217	Uman, Ukraine, 1990	JF730696	502	CC
	6 isofemale lines	Uman, Ukraine, 1990	JF730694	502	CT
	601Sin	Sinai Peninsula, Egypt, 2010	JF730694	502	CT
genotype unknown***	Z53	Zimbabwe, 1990	AF200829	14916	CT
status unknown****	Paris	Paris, France, 1952	AJ400907	14365	CT
	Astonville	New South Wales, Australia, 2002	FJ190106	12472	CT
	Brownsville	Texas, USA, 1978	FJ190107	12470	CT
	Dahomey	Benin, Africa, 1970	FJ190108	12483	CT
	Japan	Jume, Japan, 1980	FJ190109	12514	CT
	Mysore	India, Tucson Stock Centre	FJ190110	12514	CT

Notes:

* the 2757-bp sequence in JF736852 is identical to the 12508-bp sequence in FJ190105 [25];

** Oregon-R (“b” dataset) is uninfected [7], we used only information on the mitochondrial genome [39];

*** Z53 (“b” dataset) is infected [38], AF200829 [39];

**** no data on infection (all “b” dataset), sequences presented [25], [40];

LS (laboratory stock), origin unknown; repeated GenBank accession numbers JF730694 and JF730696 designate the TC and CC haplotypes; the sequence fragments of TC haplotype samples (JF730695– not indicated) were extended.

### DNA Extraction, *Wolbachia* Genotyping and mtDNA Analysis

There was one female sampled from each line and incubated in 200 µl of extraction buffer (10 mM TRIS-HCl (pH 8.0), 25 mM EDTA, 0.5% SDS, 0.1 M NaCl, 0.1 mg/ml Proteinase К) for 2 h at 56°C. The DNA was precipitated and diluted in 50 µl of deionized water. 1 µl of this solution was used for all amplifications. PCR cycling conditions were 30 cycles in 20 µl of the total volume as follows: denaturing for 5 min at 95°C; 29 cycles each for 20 s at 94°C; annealing for 1 min at 55°C (57°C for the *wsp* gene); elongation for 1 min/kbp at 72°C. The Mg^2+^ was 2.5 mM and that of each primer was 0.3 mM. The *Wolbachia* infection status was determined by amplification with the 81F/691R primer set for the *wsp* gene [16], and the 99F/994R primer set for *16SrRNA* gene [31]. The *Wolbachia* genotypes were determined by using VNTRs, IS5 and inversion markers according to the protocol [7].

We developed a system called snpPCR for detecting the 37C/T polymorphism (position 2187 in GenBank accession number NC001709), which is a diagnostic substitution for discrimination between the *M*- and *S-*clades. A search for 37C/T SNPs was carried out in two independent PCRs, one of them with COIR1 5′-CCAGTAAATAATGGGTATCAGTG-3′ and 2187-MEL 5′-GCGTTTGATTTTTTGGTGAT-3′ as primers and the other with COIR1 5′-CCAGTAAATAATGGGTATCAGTG-3′ and 2187-CS 5′-GCGTTTGATTTTTTGGTGAC-3′ as primers; 25 cycles, annealing at 55°C, Mg^2+^ at 1.5 mM. The inference about the mitotype (37C vs. 37T) depended on which of the two PCR tubes contained an amplicon. The snpPCR system was validated and verified in two ways: 1) wild-type isofemale lines infected with different genotypes and 2) 300 mutant stocks from the Laboratory of Populations Genetics, Novosibirsk, Russia (Ilinsky Yu, unpublished data). So far as the lines with *S-*clade mitotypes (**Canton-S**, **Oregon-R**, **w^1118^**, and those derived from them) often used in Drosophila labs are concerned, the 37C/T snpPCR method is a reliable technique for monitoring stock contamination as well as in *Drosophila* crossing studies.

The 2757-bp region of mtDNA contains following genes: ATPase subunits 6 and 8, three tRNAs (tRNA-Leu, tRNA-Lys, and tRNA-Asp), cytochrome c oxidase subunit II and parts of cytochrome c oxidase subunits I and III, which were sequenced using COIF 5′-CCAGCTGGAGGAGGAGATCC-3′ [32], 2672r 5′-CCAGTAAATAATGGGTATCAGTG-3′ [33], At6F 5′-GCACCTATTAGATGATTATT-3′, At6R 5′-TCGTGATACATCTCGTCATC-3′, 01 5′-TTACAAGATAGAGCTTCTCC-3′, 02 5′-ATATCATTGATGGCCGATTC-3′, 03 5′-GACGGAATTATTAAAAGTCC-3′, 04 5′-TTAGCTGTTCGATTAACTGC-3′, and COIR1 5′-GAGTTCCATGTAAAGTAGC-3′ as primers. In some lines, only 2604-bp, 1280-bp or 502-bp fragments containing relevant information were sequenced (Table 1). The 502-bp region contains two diagnostic substitutions that account for three haplogroups: CT (GenBank accession number JF730694) associated with wMel, wMel2, wMel4; CC (JF730696) – wMelCS2; and TC (JF730695) – wMelCS genotypes. The mtDNA amplicons were purified by ExoSAP-IT reagent (USB Corporation) and sequenced by BigDye® Terminator v3.1 and v1.1 cycle sequencing kits (Applied Biosystems).

**Figure 1:**
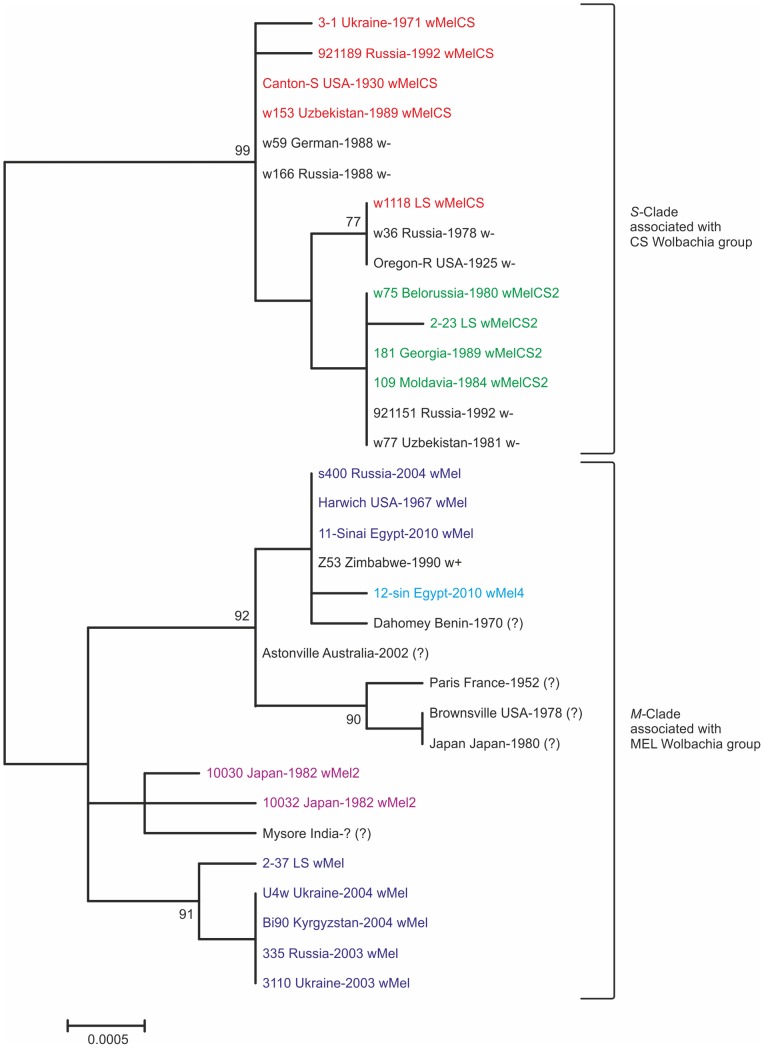
Phylogenetic tree of the 2757-bp coding-region sequence in 33 stocks (“a, b” datasets) derived from a maximum likelihood analysis of *Drosophila melanogaster* mtDNA. The tree reveals two major clades, each associated with one of the two *Wolbachia* genotype groups. Names, origin, infection status of stocks and bootstrap (1000 replicates) values higher than 75 are provided. The samples infected with identical *Wolbachia* genotypes are indicated with the same colour.

The maximum likelihood method, the Kimura 2-parameter model of nucleotide substitution [34] and 1000 bootstrap replications were used for reconstruction of the three phylogenetic trees generated by MEGA5 [35]: 1) 33 samples, alignment 2757 bp (Figure 1; “a, b” datasets); 2) 43, 1280 (Figure S1; “a, b” datasets); and 3) 327, 1515 (Figure S2; “a, b, d, e” datasets). There are no principal differences in using other methods and models. A Bayesian approach (MrBayes 3.2.1), with a general-time-reversible (GTR) model of nucleotide substitution, 1.5×10^6^ iterations of Markov chain Monte Carlo (MCMC) was used for reconstruction of phylogenetic tree with 323 samples and 2757 bp (Supporting Information S2, Supporting Information S3; “a, b, e” datasets). Posterior Bayesian probabilities were calculated on the basis of the last half of MCMC iterations.

## Results

We tried to find if there is a certain evolutionary relationship between *Wolbachia* genotypes and mtDNA diversity of *D. melanogaster*. The design of this study was to select lines with different infections status, from a broad set of locations. Coevolution changes must be observed in case of strict co-inheritance of both maternal factors: *Wolbachia* and mitochondrion. Discordance of inheritance would indicate the fact of a *Wolbachia* horizontal transmission.

The long-term association of *Wolbachia* and mitochondrion variants was investigated, “a, b, e” datasets used. Biogeography distribution of mitochondrion variants over the world was based on the analysis of “a, b, d, e” datasets. The dataset “b” was used for the analysis of *D. melanogaster* population structure and non-random distribution of the mitochondrion variants among uninfected lineages.

Each of the infected isofemale lines for which the data are provided here was unambiguously characterized by a particular *Wolbachia* genotype. In the course of these experiments, we have identified a new genotype, wMel4, in a population from the Sinai Peninsula (Egypt). It differs from the most abundant wMel genotype in wMel having seven variable number tandem repeats, VNTR-141, while wMel4 has five (Table 2). It is important to note, however, that the infection status and genotype of some stocks sequenced by other authors were unknown to us, since we used only mtDNA information available from GenBank (Table 1; “b” dataset). However, we assume the stock w^1118^, which harbors the wMelPop pathogenic strain [24], [36], [37], is infected with wMelCS. Based on genetic similarity, these *Wolbachia* genotypes fall into two groups: MEL (wMel, wMel2, wMel3, wMel4) and CS (wMelCS, wMelCS2) (Table 2).

**Table 2:** Six *Wolbachia* genotypes: genomic differences and occurrence.

Genotype group	Genotype	IS5 at WD1310 locus	IS5 at WD0516/7 locus	Number of VNTR-141 motifs	Number of VNTR-105 motifs	Inversion	Occurrence and Location*
CS	wMelCS	yes	no	6	4	forward	rare; widespread
	wMelCS2	yes	no	6	5	forward	rare; Middle Asia, East Europe, Altai
MEL	wMel	no	yes	7	5	reverse	common, widespread
	wMel2	no	yes	6	5	forward	rare; East and South Asia
	wMel3**	no	no	7	5	reverse	one laboratory stock
	wMel4***	no	yes	5	5	reverse	extremely rare; Egypt

Note:

* Data from [7], [9], [10] and original data;

** not used in this work;

*** wMel4 is a new genotype reported here.

### An Association between *D. melanogaster* mtDNA Diversity and *Wolbachia* Genotypes

The reconstructed phylogenetic tree from 33 *D. melanogaster* stocks of different origins of 2757-bp fragment of mtDNA (“a, b” datasets) reveals two main clades: *M* and *S* (the label “M” inspired by – D.**m**elanogaster and “S” – from Canton-**S**) (Figure 1, Table S1), each being strictly associated with one of the two major *Wolbachia* groups, MEL and CS respectively (Table 2). The major clades of the tree were the same when we used a shorter, 1280-bp mtDNA fragment for 43 *D. melanogaster* stocks (Figure S1; “a, b” dataset).

The mtDNA diversity in isofemale lines infected with CS genotypes is low, only 6 sites are variable. All the lines harboring wMelCS2 (derived from field collections of Eastern Europe, the Caucasus, Central Asia, and the Altai) have identical 2757-bp and identical 1280-bp sequences with the exception of laboratory stock **2–23** (origin unknown; maintained in the Laboratory of Populations Genetics since 1970), which differs from the others in only one nucleotide substitution (T → C at position 2589). The mtDNA sequences in the lines infected with the wMelCS genotype contain four variable sites. Our results show that the mitotypes associated with this genotype have no geographical pattern.

One of mtDNA variants found in MEL-infected flies is obviously widespread. The lines harboring wMel are **s400** (Sochi, the Caucasus, Russia, 2004), **Harwich (**Massachusetts, USA, 1967), **11-Sinai** (Sinai Peninsula, Egypt, 2010), and **Z53** (Zimbabwe, 1990) have identical 2757-bp mtDNA sequences. We note that **Z53** is infected, however the *Wolbachia* genotype of this strain has not been examined [38]. **12-Sin** (Sinai Peninsula, Egypt, 2010), infected with the newly discovered genotype wMel4, is quite close to the widespread mitochondrial variant of **s400**, **Harwich**, **11-Sinai**, and **Z53** lines but it differs in one substitution (A → T at position 1106). Another widespread variant occurs in Eurasia: Central Asia (Kyrgyzstan, 2004), **Bi90**; Northern Asia (Altai, Russia, 2003), **335**; and Eastern Europe (Zvenigorodka, 2003, and Uman, 2004, the Ukraine), **U4**, **3310**. Two wMel2-infected lines (both reported from Amamioshima, Japan, 1992) differ in four substitutions; however, they are most closely related to each other.

**Figure 2:**
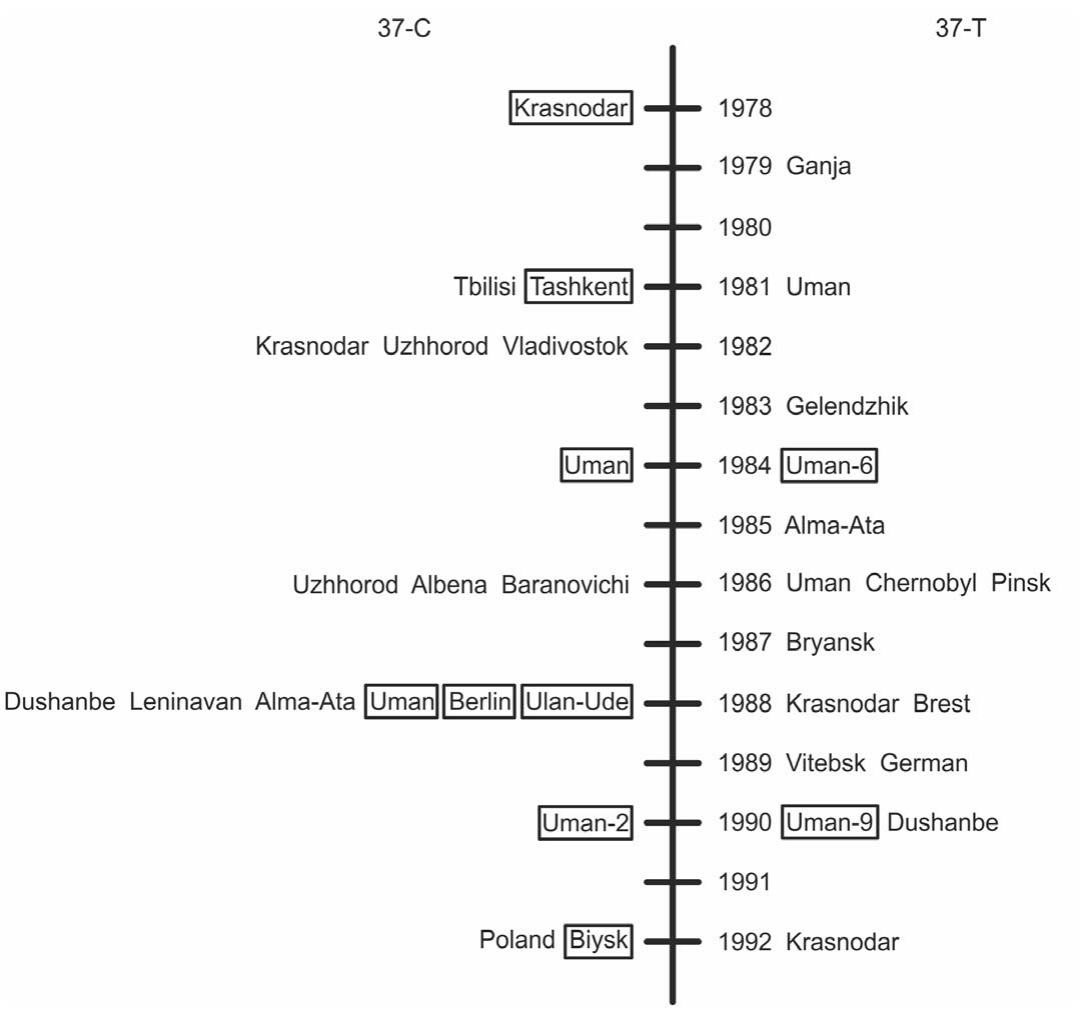
The mitochondrial clade distribution of 49 uninfected isofemale lines (“a” dataset) from North Eurasian field collections made in 1978–1992 [**9**]. This distribution was inferred using the 37C/T diagnostic substitution (37C, the *S-*clade; 37T, the *M*-clade). Boxed geographical names were confirmed by sequencing; numbers after names stand for the number of lines corresponding to a particular location.

What are relative frequencies of the *M* and *S* mitotypes in *D. melanogaster* in the wild? As far as infected flies are concerned, the answer is easy to give: the *M-*clade is the most prevalent because it is associated with the most prevalent wMel genotype. To answer this question on uninfected flies it is possible to compare 1) the ratio of *M*/*S* uninfected wild flies (Figure 2; “a, c” datasets) with that of MEL/CS genotypes and 2) the mitotype diversity of infected and uninfected flies.

On the one hand, mitotype frequencies among infected flies can be expected to differ from uninfected ones. Indeed, mitotypes of infected flies undergo an indirect selection, i.e. selection of *Wolbachia*, whereas uninfected flies are under a direct selection of mitotypes. The genetic drift leads to shift of mitotype frequencies among infected and uninfected flies. As to uninfected flies the genetic drift must be stronger then direct selection, since most mitotypes are neutral or near-neutral. On the other hand, infected lineages lose bacteria (imperfect maternal transmission) and as a result the mitotype frequencies of uninfected flies are equalized with the infected ones. Continent-island model for gene flow can be a good illustration of the case. If the mitotype ratio of uninfected flies differs from that of infected ones this means that the value of bacteria loss in flies lineages is lower than the value of selection or genetic drift for mitotypes. If ratio of uninfected flies does not differ from infected ones – there is a high rate of bacteria loss in maternal lineages. Besides, it is important to compare mitotype diversity of uninfected flies with that of infected flies. If these diversity are identical or very close that means the mutation rate is lower than that of *Wolbachia* loss.

To distinguish *M* and *S* mitotypes there have been developed the snpPCR of the 37C/T diagnostic substitution. In the uninfected isofemale lines derived from flies in the collections from North Eurasian populations in 1978–1992, 29 lines were identified as being in the *M-*clade (37C) and 20 lines as being in the *S-*clade (37T) (Figure 2). In the infected lines developed from flies of the same collections 31 lines were identified as being infected with CS genotypes and 74 lines – with a MEL group, in particular wMel genotype [9]. We performed a statistical comparison of the M/S mitotype ratio in uninfected flies and the M/S cytotype ratio (a cytotype results from a mitotype and the genotype of the infection) in infected lines. These differences are not significant (Fisher’s exact test, *p* = 0.199), which accounts for non-random sampling of uninfected lineages in populations and are likely to imply that uninfected flies had infected ancestors in the near past. In some of the uninfected lines, the 2757-bp, 1280-bp or 502-bp regions were also sequenced. The 502-bp region contains two diagnostic substitutions that account for three haplogroups: CT (GenBank accession number JF730694) associated with MEL, CC (JF730696) – wMelCS2, and TC (JF730695) – wMelCS. The result is that the infected and uninfected isofemale lines are observed to have identical sets of mtDNA variants (Figure 1, S1), which also means that *Wolbachia* infection has been recently lost from some maternal lineages of flies. The confirmation of this conclusion we find in the results of Richardson et al. [12], where the uninfected lines have the similar or identical mitotype diversity as the infected ones.

Thus the diversity and frequency of mitotypes among uninfected flies primarily depend on the gene pool of infected flies, uninfected flies replenished at the expense of bacteria loss in infected lineages.

## Discussion

### Cytotype Distribution

The analysis of *D. melanogaster* mtDNA variation and *Wolbachia* genotypes suggests a significant role for *Wolbachia* shaping in the haplotype diversity in this species. The fly cytotype is derived from the mitotype and infection status. Each of the two mitochondrial clades is associated with one of the two *Wolbachia* genotype groups: *M*, with MEL, and *S*, with CS. There are four different cytotypes in the wild: *M*-MEL, *M*-w^−^, *S*-CS and *S*-w^−^; however, their relative frequencies are not equal. *M*-MEL and *M*-w^−^ collectively make up about 90% or more, while *S*-CS and *S*-w^−^ –10% or less.

#### Genotype distribution

A high frequency of the wMel genotype was reported previously [7]–[10], [12]. A few wMelCS cases are known; however, they were reported from different regions of the world [7]. The wMelCS2 genotype is likely to be limited to *D. melanogaster* populations in Eastern Europe, the Caucasus, Central Asia, and the Altai [7]–[9]. The wMel2 genotype has been found in Japan, China, India and Southeast Asia [7], [10], while wMel3, in just one *D. melanogaster* stock kept under laboratory conditions [7]. Additionally, we have found a new genotype, wMel4, in a population from the Sinai Peninsula (Egypt). Nunes et al. [10] also reported a new genotype from Uganda, the latter genotypically belongs to the MEL group; however, it is not named and there are no data on the status of its inversion marker.

#### Mitotype distribution

There are data [8], [10], [12] that most uninfected flies have the same mitotypes as flies infected with wMel genotype. Furthermore, it is possible to make inferences about *M*- and *S*-clade frequencies from the data on mitochondrial sequences in lines that were studied without reference to *Wolbachia* whatsoever. Previous studies [12], [23], [29], [39] (“b, d, e” datasets) have focused on *M*-clade genomes. Seven complete or nearly complete mtDNA genomes presented in GenBank belong to the *M-*clade (AJ400907, AF200829, FJ190106–10) and only two sequences belong to the *S-*clade (AF200828 and FJ190105) (Table 1) [25], [39], [40]. The genetic distance ranges from 0.0002 to 0.0019 within the *M-*clade and it is 0.0001 within the *S*-clade; while between these two clades it ranges from 0.0037 to 0.0042.

**Figure 3:**
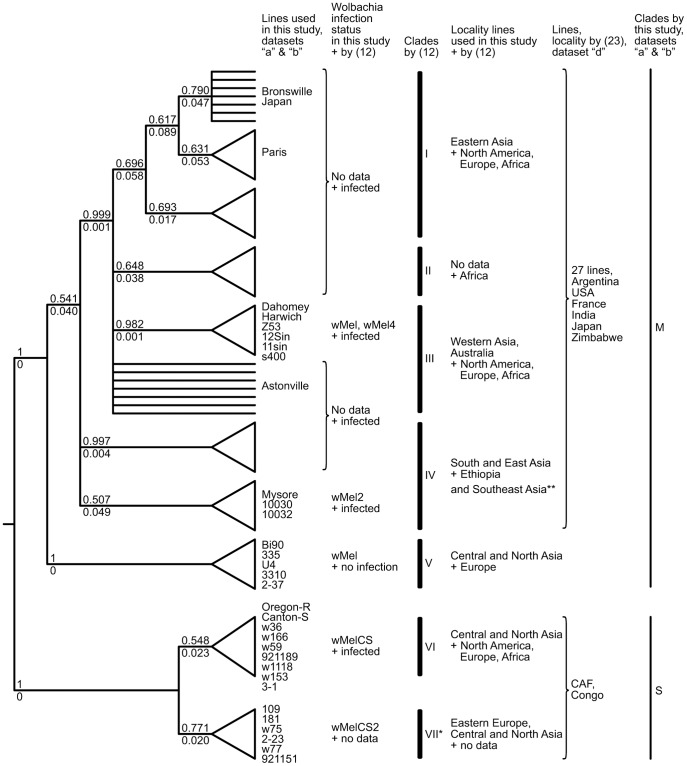
Schematic tree of 2757 bp mtDNA based on Bayesian genealogy of 323 *Drosophila melanogaster* lines (“a, b, e” datasets) from over the world (Supporting Information S3). Posterior Bayesian probabilities and SE indicated above and below nodes, respectively. Shown are 1) the position of our lines and those of [25], [39], [40] in the tree and their associations with *Wolbachia* genotypes; 2) the correspondence between clades identified in Richardson et al. [12] and our datasets; clade VII*, identified here by us, is associated with wMelCS2 genotype; 3) the biogeography data of mitotypes; Southeast Asia** according to [10] where genotype wMel2 was found; 4) 28 samples from Rand et al. [23] (“d” dataset) belonging to the tree that is based on the analysis of relevant region of 1515 bp alignment (Supporting Information S1). Clade-branch I-II-III-IV is not clearly resolved in this case (Figure S2); 5) the correspondence of the tree to *M* and *S* clades.

Findings of the recently performed study [12] in which complete *Wolbachia* and mtDNA genome sequences of 290 *D. melanogaster* lines were presented confirm our results: in particular they show a strict concordance between *Wolbachia* and mtDNA lineages. The most lines in this study also belong to the *M*-clade, 285 versus 5 of the *S*-clade. The detailed analysis [12] made it possible to subdivide *M*-clade into 5 clades (I–V) and to refer our S-clade to their VI-clade. A combined analysis of mtDNA from [12], [23] and our data allow us to get a more comprehensive picture of the biogeography of mitotypes all over the world. The phylogenetic tree for 323 lines (290 of “e” +33 from “a, b” datasets) for 2757 bp alignment distinguishes all 6 clades (Figure 3, Supporting Information S2). In addition to clades I–VI there is a new clade VII that is associated with wMelCS2 genotype. Clade V in the original study [12] was not associated with *Wolbachia* and it was found in a very small number of stocks. Our results show that clade V is associated with the wMel genotype and its mitotypes are widespread in Eurasia. The samples that belong to clade IV in particular are associated with wMel2 (Figure 1, 3). Based on distribution of wMel2 genotype [7], [10] these mitotypes spread not only in Africa [12] but over South, East and Southeast Asia. The samples of clade II are absent in our collection and according to [12] they are limited within Africa. Clades I and III are evidently the most spread over the world. In addition to mitotype distribution of “a, b, e” datasets we compared 1515 bp mtDNA of 27 isofemale lines (“d” dataset) from Africa, Eurasia and the Western hemisphere from [23] with the relevant genome information. The CAF line from Congo is the most close to *S*-clade whereas other lines (USA, Argentina, France, India, Japan, Zimbabwe) cluster with the I-II-III-IV clade-branch, which is not clearly resolved for 1515 bp analyzed region (Supporting Information S1).

### *S/M* Clade Divergence

Tropical Africa is the ancestral range of *D. melanogaster* [41]. This conclusion was made on the basis of allozymic variability analysis [42], nuclear gene sequences [43]–[53] and mtDNA restriction-site polymorphism [54]. The world regions where *D. melanogaster* populations reside were subdivided into three categories: ancestral regions (tropical Africa), ancient regions (Eurasia) and new regions (Australia and the Western Hemisphere) [41], [54].

In light of the current *D. melanogaster* biogeography, it is interesting to ask whether mtDNA diversity has evolved in the African populations or after *D. melanogaster* had spread over the world? *D. melanogaster* migration from Africa to Eurasia might begin after the last glaciations [41], [44]–[50], 10–12 thousand years ago, and it has spread with advancements in agriculture and, in recent centuries, with the European colonization of Australia and the Americas. The simple evidence of the African origin of the mitotype diversity is the presence of samples of I-II-III-IV- (*M-*) and of VI- (*S-*) clades and the absence only of V- (*M-*) and VII- (*S-*) ones in Africa. In addition, to address this question, we calculated the possibly most recent *S/M-*clade divergence time based on one of the values obtained by direct estimation of the *D. melanogaster* mtDNA mutation rate [55]. The mitochondrial genome of Oregon-R (14905 bp, AF200828) belongs to the *S-*clade, and that of Z53 (14916 bp, AF200829), to the *M-*clade. These genomes differ in 52 single nucleotide mutations and four indels. Assuming that the average mutation rate for every type of a single substitution and an indel is 9.2×10^−8^ per site per generation [55] and that *D. melanogaster* has up to with 20 generations per year in the wild, the time required for this number of mutations to happen in two mitochondrial molecules is more than 1000 years. Of course such value of diversification is too much underestimated because it considers only the fact of mutation but not of fixation in mitochondrial population of the individual. Moreover, it is obvious that reverse and repeat mutations could happen in Oregon-R and Z53 ancestors. It means that the *S-* and *M-*clades had diverged long before enhanced human activity promoted the *D. melanogaster* spread over the Earth and probably even before the end of the last glacial period. It is necessary to note Richardson et al [12] also conclude that the origin of the global cytoplasmic diversity is in Africa based on a Bayesian phylogenetic analysis. Moreover the estimation of divergence of *Wolbachia* that are associated with *M*- and *S*-clade is 3263–13998 ya [12] which supports our conclusion on *M-* and *S-*clade divergence in Africa. If that scenario is true, then the place where *Wolbachia* diverged into two genotype groups was Africa, while the wMelCS2 and wMel2 genotypes are likely to have originated in the regions where they were found: Middle Asia and Eastern Europe (wMelCS2) and South, East and Southeast Asia (wMel2). However, it is quite possible that wMel2 exists in Africa because IV-clade mtDNA was found there.

### A Hypothesis of Global *Wolbachia* Replacement

Reigler et al. [7] suggested the hypothesis of global *Wolbachia* replacement in *D. melanogaster*. Their hypothesis is based on the fact that wMelCS was originally present in field collections made before the 1970’s, and later the wMel genotype became dominant. However, the number of wMelCS-infected isofemale lines attributed to the middle of the 20^th^ century is small (n = 14).

The global *Wolbachia* replacement should result in changes in the mitochondrial variation pattern in uninfected flies, if *Wolbachia* transmission is imperfect and there is no horizontal transfer. Identical mitochondrial variants have been found in both infected and uninfected *D. melanogaster*, which is indicative of a recent loss of infection in maternal lineages. Since selection favors infected females, the number of uninfected ones decreases over time. Consequently, the frequency of *M-*clade occurrences should increase and that of *S-*clade occurrences should decrease in both infected and uninfected lineages. Nunes et al. [10] attempted to verify this hypothesis by comparing the ratio of different mitotypes in 10 long-standing isofemale lines (derived before 1955) with the mitochondrial pattern as in modern field collections. Following [10], we tracked the *M-* and *S-*clade dynamics in uninfected isofemale lines by PCR screening for the presence of the 37C/T polymorphism. We found that a considerable number of uninfected flies belonging to the *S-*clade existed in North Eurasian populations in 1978–1992 (see Results and Figure 2). Therefore, a big contribution to the *S-*clade lineages that come from the wMelCS-infected cytoplasm (the TC mitotype, JF730695) has been made by lineages that used to harbor the wMelCS2 infection (the CC mitotype, JF730696; they both have 37C), that is confined to Northern Eurasia [7], [9]. This implies that all other regions of the world could be characterized at that period by fewer *S*-clade occurrences, and as a consequence a replacement of cytotypes is driven at a different rate in different regions of the world.

Richardson et al. [12] came to the conclusion that replacement of genotypes is incomplete and it began long before the 20^th^ century, which is confirmed by large *M*-clade diversity (Figure 3, Supporting Information S3). So the most intriguing question is what is the cause of a notable number of wMelCS laboratory stocks established at the first half of the last century. Further analyses of the *S-* and *M-*clade dynamic among uninfected lines is needed to clarify the scale and rate of replacement events.

### Horizontal Transmission of *Wolbachia*

Neither we nor [12] found evidence for horizontal transmission of MEL or CS genotypes between the clades, it is still possible for such events to occur in the wild. They can be detected by mere comparing diagnostic SNPs in infected flies; however, a low frequency of non-MEL genotypes in field populations poses a challenge.

No detection of *Wolbachia* strains in *D. melanogaster* other than those related to the wMel strain were reported earlier. Admittedly, several “undetermined genotypes” of *Wolbachia* in *D. melanogaster* were reported [10]. These isolates did not amplify the VNTR-141, VNTR-105 or IS5-WD0615/7 markers; however, they amplified with IS5-WD1310 and looked very similar to the MEL-group entities. Although we have not observed such *Wolbachia* genotyping profile in *D. melanogaster*, we have seen it in a different *Drosophila* species from Thailand, in which the *cox1* gene has a high similarity with that in *D. ananassae, D. pallidosa* and *D. papuensis* (results not shown). With the exception of any possible methodological difficulties of genotyping, the origin of such “undetermined genotypes” might be accounted for by horizontal transmission of *Wolbachia* from a different host.

In summary, the modern mitochondrial pattern in *D. melanogaster* is characterized by a low variation possibly resulting from a selective sweep of *Wolbachia*. The cytotypes occur at different frequencies: individuals with *M-*clade cytotypes are most prevalent in the populations of the world. The remainder of individuals should be in the *S-*clade, although existence of some clades more is but not excluded, for instance, in tropical Africa. Uninfected and *Wolbachia-*infected flies have identical sets of mitotypes within each of the *M-* and *S-*clades. This is a likely indication of a recent infection loss from maternal lineages and an important contribution of *Wolbachia* selection to the mtDNA pattern of variation in *D. melanogaster*.

## Supporting Information

Figure S1 **Phylogenetic tree of the 1280-bp coding-region sequence in 43 stocks, derived from a maximum likelihood analysis of** ***Drosophila melanogaster*** **mtDNA.** Names, origin, infection status of stocks and bootstrap (1000 replicates) values higher than 75 are provided. The samples infected with identical *Wolbachia* genotypes are indicated with the same colour.
(TIF)

Figure S2 **Phylogenetic tree of the 1515 bp alignment in 327 stocks (“b, d, e” datasets), derived from a maximum likelihood analysis of** ***Drosophila melanogaster*** **mtDNA.**
(TIF)

Table S1 **Nucleotide polymorphism in 2757 bp mtDNA of 33** ***Drosophila melanogaster*** **lines (“a, b” datasets).**
(DOC)

Supporting Information S1 **Archive of the 1515 bp alignment extracted from** [**12**], [23], [25], [39], [**40**] **(“b, d, e” datasets) in Fasta format.**
(FASTA)

Supporting Information S2 **Archive of the 2757 bp alignment extracted from** [**12**], [25], [39], [**40**] **(“a, b, e” datasets) in Fasta format.**
(FASTA)

Supporting Information S3 **Phylogenetic tree of 323 samples of 2757 bp alignment in Nexus format.** A Bayesian approach (MrBayes 3.2.1), with a general-time-reversible (GTR) model of nucleotide substitution, 1.5×10^6^ iterations of MCMC was used.
(TRE)
