# Supplementary figures and images for: Coevolution of Drosophila melanogaster mtDNA and Wolbachia Genotypes
Source: PLoS One. 2013 Jan 17;8(1):e54373. doi: 10.1371/journal.pone.0054373 (PMC3547870; doi:10.1371/journal.pone.0054373)

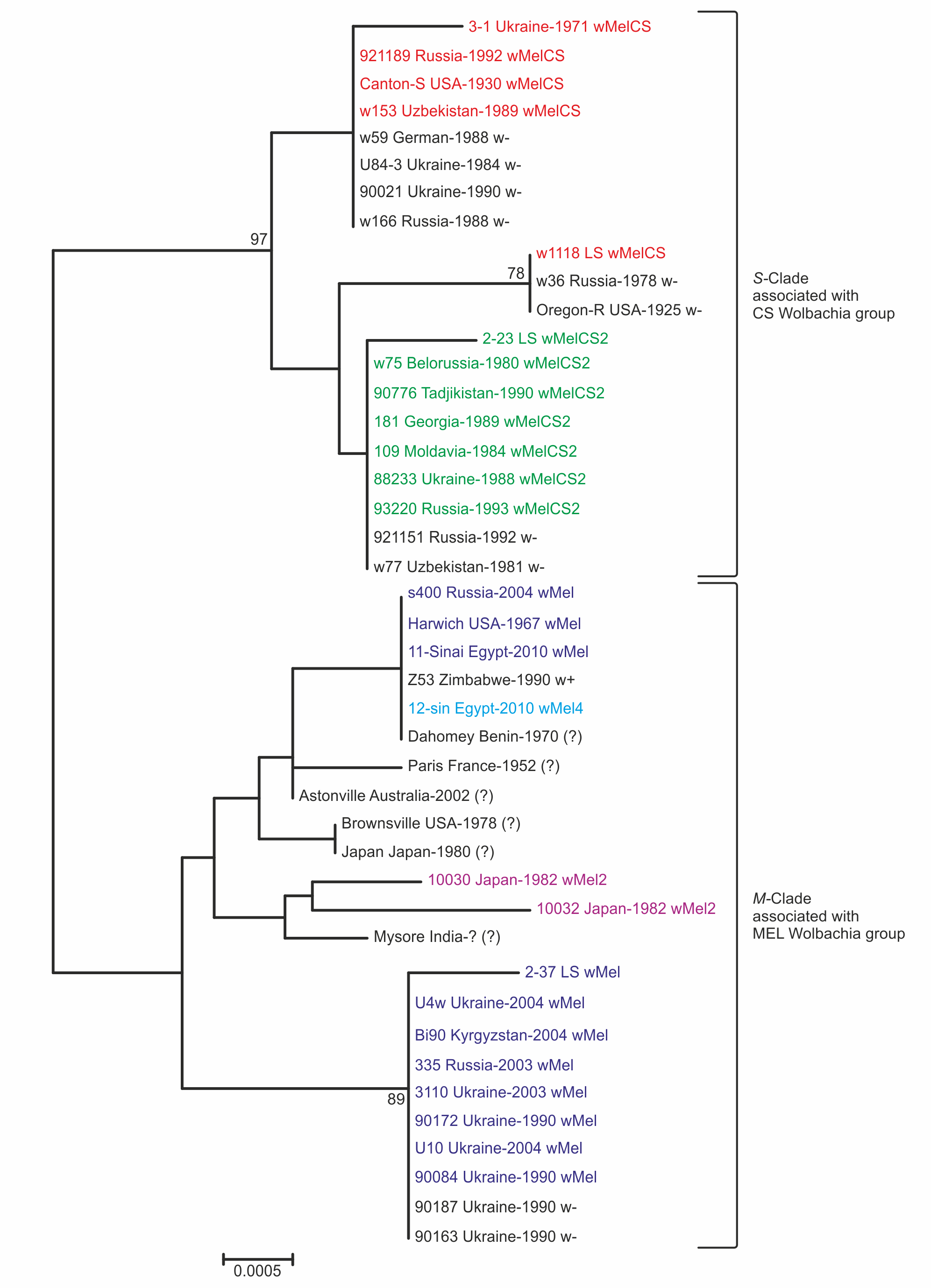

Supplement: Figure S1 — Phylogenetic tree of the 1280-bp coding-region sequence in 43 stocks, derived from a maximum likelihood analysis of Drosophila melanogaster mtDNA. Names, origin, infection status of stocks and bootstrap (1000 replicates) values higher than 75 are provided. The samples infected with identical Wolbachia genotypes are indicated with the same colour. (TIF) [file pone.0054373.s001.tif]

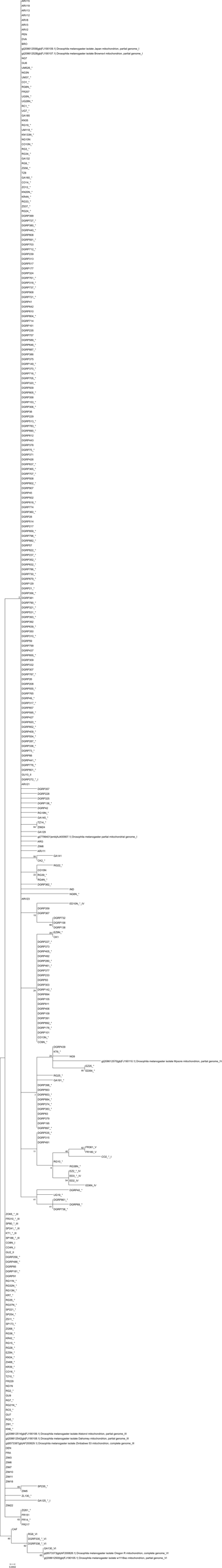

Supplement: Figure S2 — Phylogenetic tree of the 1515 bp alignment in 327 stocks (“b, d, e” datasets), derived from a maximum likelihood analysis of Drosophila melanogaster mtDNA. (TIF) [file pone.0054373.s002.tif]
