# Supplementary material for: Coevolution of Drosophila melanogaster mtDNA and Wolbachia Genotypes
Source: PLoS One. 2013 Jan 17;8(1):e54373. doi: 10.1371/journal.pone.0054373 (PMC3547870; doi:10.1371/journal.pone.0054373)
Supplement: Table S1 — Nucleotide polymorphism in 2757 bp mtDNA of 33 Drosophila melanogaster lines (“a, b” datasets). (DOC) [file pone.0054373.s003.doc]

| Stock, Wolbachia infection, GenBank accession | 1  0 | 3  7 | 2  5  9 | 5  1  1 | 8  1  4 | 1  0  4  7 | 1  1  0  6 | 1  3  6  6 | 1  4  3  3 | 1  5  3  5 | 1  5  5  6 | 1  8  7  3 | 1  9  3  8 | 1  9  4  4 | 2  0  3  7 | 2  0  9  7 | 2  1  6  9 | 2  2  7  1 | 2  2  7  7 | 2  4  4  2 | 2  4  4  9 | 2  4  6  6 | 2  4  7  0 | 2  5  2  4 | 2  5  4  1 | 2  5  8  9 | 2  6  1  2 | 2  7  0  3 | 2  7  1  8 |
| --- | --- | --- | --- | --- | --- | --- | --- | --- | --- | --- | --- | --- | --- | --- | --- | --- | --- | --- | --- | --- | --- | --- | --- | --- | --- | --- | --- | --- | --- |
| **consensus** | C | T | T | C | G | G | A | C | T | A | G | T | C | C | C | C | A | T | A | T | T | A | G | G | T | T | C | G | G |
| 921151, w-, JF736863 |  | C |  |  |  |  |  | T |  | G |  |  | T |  |  | A |  |  |  |  |  |  | A |  |  |  |  |  |  |
| w77, w-, JF736861 |  | C |  |  |  |  |  | T |  | G |  |  | T |  |  | A |  |  |  |  |  |  | A |  |  |  |  |  |  |
| 2-23, wMelCS2, JF736864 |  | C |  |  |  |  |  | T |  | G |  |  | T |  |  | A |  |  |  |  |  |  | A |  |  | C |  |  |  |
| 181, wMelCS2, JF736851 |  | C |  |  |  |  |  | T |  | G |  |  | T |  |  | A |  |  |  |  |  |  | A |  |  |  |  |  |  |
| 109, wMelCS2, JF736850 |  | C |  |  |  |  |  | T |  | G |  |  | T |  |  | A |  |  |  |  |  |  | A |  |  |  |  |  |  |
| w75, wMelCS2, JF736846 |  | C |  |  |  |  |  | T |  | G |  |  | T |  |  | A |  |  |  |  |  |  | A |  |  |  |  |  |  |
| Oregon-R, w-, AF200828 | T | C |  |  |  |  |  | T |  | G |  |  | T |  |  | A |  |  |  | A |  |  | A |  |  |  |  |  |  |
| w^1118^, wMelCS, JF736852 | T | C |  |  |  |  |  | T |  | G |  |  | T |  |  | A |  |  |  | A |  |  | A |  |  |  |  |  |  |
| w36, w-, JF736859 | T | C |  |  |  |  |  | T |  | G |  |  | T |  |  | A |  |  |  | A |  |  | A |  |  |  |  |  |  |
| 921189, wMelCS, JF736847 | T | C |  |  |  |  |  | T |  | G |  |  |  |  |  | A |  |  |  |  |  |  | A |  |  |  |  |  | A |
| 3-1, wMelCS, JF736867 | T | C | C |  |  |  |  | T |  | G |  |  |  |  |  | A |  |  |  |  |  |  | A |  |  |  |  |  |  |
| w153, wMelCS, JF736849 | T | C |  |  |  |  |  | T |  | G |  |  |  |  |  | A |  |  |  |  |  |  | A |  |  |  |  |  |  |
| w59, w-, JF736860 | T | C |  |  |  |  |  | T |  | G |  |  |  |  |  | A |  |  |  |  |  |  | A |  |  |  |  |  |  |
| w166, w-, JF736862 | T | C |  |  |  |  |  | T |  | G |  |  |  |  |  | A |  |  |  |  |  |  | A |  |  |  |  |  |  |
| Canton-S, wMelCS, JQ416156 | T | C |  |  |  |  |  | T |  | G |  |  |  |  |  | A |  |  |  |  |  |  | A |  |  |  |  |  |  |
| U4w, wMel, JF736845 |  |  |  |  |  |  |  |  |  |  | A |  |  | T |  |  |  |  |  |  |  |  |  |  | C |  |  |  |  |
| Bi90, wMel, JF736853 |  |  |  |  |  |  |  |  |  |  | A |  |  | T |  |  |  |  |  |  |  |  |  |  | C |  |  |  |  |
| 335, wMel, JF736854 |  |  |  |  |  |  |  |  |  |  | A |  |  | T |  |  |  |  |  |  |  |  |  |  | C |  |  |  |  |
| 3110, wMel, JF736855 |  |  |  |  |  |  |  |  |  |  | A |  |  | T |  |  |  |  |  |  |  |  |  |  | C |  |  |  |  |
| 2-37, wMel, JF736858 |  |  |  |  |  |  |  |  |  |  |  | C |  | T |  |  |  |  |  |  |  |  |  |  | C |  |  |  |  |
| Z53, w+(?), AF200829 |  |  |  |  | A |  |  |  |  |  |  |  |  |  |  |  |  |  |  |  | C | T |  |  |  |  | T |  |  |
| s400, wMel, JF736848 |  |  |  |  | A |  |  |  |  |  |  |  |  |  |  |  |  |  |  |  | C | T |  |  |  |  | T |  |  |
| Harwich, wMel, JF736865 |  |  |  |  | A |  |  |  |  |  |  |  |  |  |  |  |  |  |  |  | C | T |  |  |  |  | T |  |  |
| 11-Sinai, wMel, JF781531 |  |  |  |  | A |  |  |  |  |  |  |  |  |  |  |  |  |  |  |  | C | T |  |  |  |  | T |  |  |
| 12-Sin, wMel4, JF736866 |  |  |  |  | A |  | T |  |  |  |  |  |  |  |  |  |  |  |  |  | C | T |  |  |  |  | T |  |  |
| Dahomey, ?, FJ190108 |  |  |  |  | A |  |  |  |  |  |  |  |  |  |  |  |  |  |  |  | C | T |  |  |  |  | T | A |  |
| Astonville, ?, FJ190106 |  |  |  |  | A |  |  |  |  |  |  |  |  |  |  |  |  |  |  |  | C |  |  |  |  |  | T |  |  |
| Brownsville, ?, FJ190107 |  |  |  | T | A |  |  |  | C |  |  |  |  |  |  | T |  |  |  |  | C |  |  |  |  |  | T |  |  |
| Japan, ?, FJ190109 |  |  |  | T | A |  |  |  | C |  |  |  |  |  |  | T |  |  |  |  | C |  |  |  |  |  | T |  |  |
| Paris, ?, AJ400907 |  |  |  | T | A |  |  |  | C |  |  |  |  |  |  |  |  |  | G |  | C |  |  |  |  |  | T |  |  |
| Mysore, ?, FJ190110 |  |  |  |  |  | A |  |  |  |  |  |  |  |  | T |  |  |  |  |  | C |  |  |  |  |  |  |  |  |
| 10030, wMel2, JF736856 |  |  |  |  |  |  |  |  |  |  |  |  |  |  | T |  | G |  |  |  |  |  |  |  |  |  |  |  |  |
| 10032, wMel2, JF736857 |  |  |  |  |  |  |  |  |  |  |  |  |  |  | T |  |  | C |  |  |  |  |  | A |  |  |  |  |  |
